# Association between occupational stress, markers of inflammation, and oxidative stress in intensive care unit workers: a cross-sectional study

**DOI:** 10.47626/1679-4435-2022-1049

**Published:** 2024-09-24

**Authors:** Luciano Antonio Rodrigues, Adriene de Freitas Moreno Rodrigues, Manuela Negrelli Brunetti, Monique Michels, Mariane Abatti, Amanda Indalécio, Luana Bezerra Rocha, Felipe Dal Pizzol

**Affiliations:** 1 Ciências da Saúde, Centro Universitário do Espírito Santo, Colatina, ES, Brasil; 2 Ciências da Saúde, Universidade do Extremo Sul Catarinense, Criciúma, SC, Brasil

**Keywords:** burnout, occupational stress, health professional, oxidative stress, inflammation, esgotamento profissional, estresse oxidativo, estresse ocupacional

## Abstract

**Introduction:**

Health care workers in intensive care units have a high propensity to develop burnout syndrome.

**Objectives:**

To evaluate the relationship between occupational stress and markers of oxidative stress and inflammation in health care workers in intensive care units.

**Methods:**

The sample consisted of 133 intensivists from the city of Colatina, state of Espírito Santo, Brazil. The Maslach Inventory Burnout Survey was used to assess burnout syndrome. Oxidative stress was measured in proteins and lipids, and cytokine levels were assessed via enzyme-linked immunosorbent assay.

**Results:**

The highest levels of burnout syndrome (emotional exhaustion dimension) were found in nurses and physical therapists and showed greater changes in markers of protein damage and inflammation. On the emotional exhaustion dimension, it was higher among professionals who consumed some type of alcoholic beverage and some type of stimulant, whether caffeine, tea, or soft drinks, at least twice a week. There was a positive relationship between the development of burnout syndrome, specifically in the dimension of low personal involvement at work, and oxidative damage in lipids (thiobarbituric acid reactive substances).

**Conclusions:**

There is evidence of relationship between occupational stress and oxidative stress in professionals with low personal involvement in their work.

## Abstract (pt)

**Introdução:**

Os profissionais de saúde que atuam em Unidades de Terapia Intensiva apresentam forte propensão a desenvolver a síndrome de *burnout.*

**Objetivos:**

Este estudo teve como objetivo avaliar a relação entre o estresse ocupacional e os marcadores de estresse oxidativo e inflamação em profissionais de saúde em Unidade de Terapia Intensiva.

**Métodos:**

A amostra foi composta por 133 profissionais intensivistas de Colatina, Espírito Santo, Brasil. O Maslach Inventory Burnout Survey foi aplicado para avaliar a síndrome de *burnout.* O estresse oxidativo em proteínas e lipídios foi medido e os níveis de citocinas foram avaliados pelo ensaio *enzyme-linked immunosorbent assay.*

**Resultados:**

Os resultados do estudo mostraram que os níveis mais elevados para síndrome de *burnout* (dimensão exaustão emocional) foram mais significativos entre enfermeiros e fisioterapeutas e apresentaram maiores alterações nos marcadores de dano proteico e inflamação. Na dimensão exaustão emocional, foi maior nos profissionais que consomem algum tipo de bebida alcoólica pelo menos duas vezes por semana e algum tipo de estimulante, seja cafeína, chá ou refrigerante. Houve relação positiva no desenvolvimento da síndrome de *burnout,* na dimensão de baixo envolvimento pessoal no trabalho, com dados oxidativos em lipídios (*thiobarbituric acid reactive substances*).

**Conclusões:**

Há evidências de relação entre estresse ocupacional e estresse oxidativo em profissionais com baixo envolvimento pessoal em seu trabalho.

## INTRODUCTION

Globalization has characteristics that are present in different landscapes of society, including occupational health. The quality of new high-stress functions in an environment of business continuity and technological capability. Because of the demands and the search for change in the organizational environment, adapting to this new setting has a significant impact on the health of workers, triggering psychological disorders, cognitive wear, psychological suffering, and suffering at work.^1^

Stress has been studied in several professions, and health care workers have been found to be particularly susceptible to physical and emotional exhaustion. These professionals are under constant pressure to be productive, especially when there is an imbalance between their work activities and the expectations placed on them.^2,3^

Factors identified as triggers of stress among health care workers include the need to deal with suffering, pain and death, low pay, job insecurity, excessive working hours and night shifts, risk of occupational disease, lack of resources to perform their duties, and other related issues.^4^

### EMPLOYEE HEALTH AND OCCUPATIONAL STRESS

Work must be recognized as a source of individual satisfaction. In this context, it is necessary to balance professional activities in order to achieve well-being. Quality of life is directly linked to the individual’s working environment, and joint efforts are needed to improve working conditions in order to maintain the physical and mental health of workers.^5^

Under these circumstances, the word “stress” has become commonplace in everyday life, often associated with the intensification of work-related problems, and is particularly prevalent in health care settings. A Chinese study with more than 650 medical teams found professional burnout to be very high among health care workers. Factors that increase stress included the work environment and aspects related to the professionals’ personalities, particularly their low self-esteem and how they deal with daily problems.^6^

In recent decades, research has focused on the occupational health of professionals in various fields. Among these studies, a syndrome has been identified that has been characterized as an occupational disease called burnout syndrome. “Burnout” refers to something that no longer functions because of exhaustion. Some authors describe burnout as becoming exhausted after excessive demands on energy or strength.^7,8,9^

In the new International Statistical Classification (lCD-11) (2022) , burnout syndrome receives a more specific definition and is categorized under problems associated with employment or unemployment. It has been assigned a specific code, QD85, with the spelling “burn-out”. This refers to a syndrome that is conceptualized as being caused by phenomena in the occupational context, and it has three components that are conceptualized in a multidimensional way.^10^ At the 72nd World Health Assembly in Geneva at the end of May 2019, burnout was confirmed as a syndrome resulting from chronic work-related stress. The new classification establishes a standardized language to facilitate the exchange of information on the topic among health care workers worldwide.^11^

### BURNOUT, OXIDATIVE STRESS, AND INFLAMMATION

Work-related stressors that cause pathological signs and symptoms are important in identifying burnout syndrome, or professional exhaustion syndrome. These terms are synonymous and manifest as psychosomatic, psychological, and social symptoms resulting from excessive workload over an extended period of time.^12^ The most commonly cited definition of this condition comes from Maslach & Jackson,^13^ who describe burnout as a syndrome characterized by emotional exhaustion, depersonalization, and diminished professional accomplishment that affects individuals who work with people.

Psychological changes can cause damage to the body, and oxidative damage induced in cells and tissues has been implicated in the etiology of several diseases, including degenerative conditions (e.g., ischemic heart disease and diabetes). Inflammatory processes increase the production of reactive oxygen species (ROS), leading to disorders such as endothelial dysfunction.^14^ Oxidative stress can induce an inflammatory process, and excessive inflammation can cause oxidative stress, resulting in significant cellular damage and tissue destruction. Therefore, we are evaluating the relationship between occupational stress, inflammation, and oxidative stress in professionals working in intensive care units (ICUs).

## METHODS

This study was conducted with health care workers from intensive care units of a hospital in the city of Colatina, state of Espírito Santo (ES), Brazil. Initially, we used a qualitative approach to identify the sociodemographic profile, professional characteristics, and lifestyle of the participants.

The characteristics of occupational stress identified as burnout syndrome were assessed by using the Maslach Burnout Inventory and Human Services Survey (MBI-HSS).^13^ In addition, a quantitative assessment of oxidative stress markers for lipid damage (thiobarbituric acid reactive substances [TBARs]), protein damage (carbonyl), and inflammation markers interleukin (IL)-6 and IL-10 was performed. Sample size was calculated considering a power of 80 and an alpha error of 0.05, resulting in a sample size of 133.

Inclusion criteria were health care workers from adult ICUs in hospitals with larger patient flows, those employed for at least 6 months, and those with a work history that included more than one job. Exclusion criteria were health care workers who worked in other sectors of the same hospital, those who participated in only one aspect of the research (either interview or blood sampling), ICU staff on sick leave, those who died, and those who refused to participate. As a result, 33 professionals were excluded: 22 left the service or sector during the study, 3 were excluded due to maternity leave, 7 withdrew during blood sampling, and 1 died during the study period.

A cross-sectional epidemiological survey was conducted in two parts. In the first part, interviews were conducted using two data collection instruments: 1) one to determine the sociodemographic profile of the professionals, their professional characteristics and lifestyle; and 2) the other to determine the presence and extent of burnout according to its dimensions and levels. In the second part of the study, blood samples were drawn in order to assess inflammation and oxidative stress.

The MBI-HSS^5^ was used to assess burnout. The Brazilian version of the questionnaire contains 22 items divided into three dimensions: emotional exhaustion, depersonalization, and reduced personal accomplishment. The inflammatory response markers (i.e., IL-6 and IL-10 levels) were measured using a commercial kit (R&D Systems). Oxidative damage was analyzed by lipid peroxidation through the formation of thiobarbituric acid reactive substances (TBARS).^15^ Protein oxidative damage was assessed by determining the carbonyl groups in the sample based on their reaction with dinitrophenylhydrazine.^16^

Continuous variables are presented as mean ± standard deviation (SD) and were compared using the Kruskal-Wallis test as they were not normally distributed. Qualitative variables are presented as number (percentage) N (%) and were compared using the chi-square test followed by residual analysis. All tests were analyzed using Statistical Packages for the Social Sciences (SPSS) version 21. In all analyses, a p < 0.05 was used as the level of statistical significance. This study was approved by the Research Ethics Committee of Centro Universitário do Espírito Santo (UNESC), city of Colatina, state of Espírito Santo, under no. 61075716.4.0000.5062.

## RESULTS

The sociodemographic profile of the ICU workers studied included a total of 133 intensivists from two public hospitals with ICUs for the treatment of adult patients, who were included in the study between February 2017 and June 2018. Five professional categories participated in the study: 19 nurses, 13 physical therapists, 3 speech therapists, 26 physicians, and 72 nursing technicians. Nursing technicians made up most of the participants (54.1%), followed by physicians, nurses, physical therapists, and speech therapists.

Nursing staff consisted of two types of professionals (nurses and nursing technicians) and represented 68.4% of the sample. Participants were aged 21-72 years (35.1 ± 9.5 years). Most participants were women (64.6%), married (49.6%), aged 26-41 years (63.9%), and had lived in the community for more than 15 years (46.6%). Half of them had children (51.1%), of whom 42.9% had either one or two children.

Table 1 shows the sociodemographic profile of the ICU workers by category and their relationship with the three dimensions and levels of burnout: emotional exhaustion, depersonalization, and decreased personal accomplishment. High levels of burnout were found in the emotional exhaustion dimension of the sample, which was statistically significant for nurses (78.9%) and physical therapists (76.9%). Nursing technicians had the lowest levels of emotional exhaustion and depersonalization (54.2% and 77.8%, respectively).

**Table 1:** Sociodemographic profile of ICU workers according to the dimensions and levels of burnout syndrome

Variables	Dimensions and levels of burnout syndrome								
	Emotional exhaustion			Depersonalization			Personal accomplishment		
	Low n (%)	High n (%)	p-value	Low n (%)	High n (%)	p-value	Low	High n (%)	p-value
Professional category									
Nursing technicians	39 (54.2)	33 (45.8)	0.008	56 (77.8)	16 (22.2)	0.082	56 (77.8)	16 (22.2)	0.526
Nurses	4 (21.1)	15 (78.9)		10 (52.6)	9 (47.4)		10 (52.6)	9 (47.4)	
Physicians	13 (50.0)	13 (50.0)		15 (57.7)	11 (42.3)		15 (57.7)	11 (42.3)	
Physical therapists	3 (23.1)	10 (76.9)		8 (61.5)	5 (38.5)		8 (61.5)	5 (38.5)	
Speech therapists	0 (0.0)	3 (100.0)		1 (33.3)	2 (66.7)		1 (33.3)	2 (66.7)	
Sex									
Male	17 (36.2)	30 (63.8)	0.160	28 (59.6)	19 (40.4)	0.140	28 (59.6)	19 (40.4)	0.532
Female	42 (48.8)	44 (51.2)		62 (72.1)	24 (27.9)		62 (72.1)	24 (27.9)	
Age, years									
18-25	10 (47.6)	11 (52.4)	0.639	18 (85.7)	3 (14.3)	0.312	18(85.7)	3 (14.3)	0.160
26-33	17 (37.8)	28 (62.2)		30 (66.7)	15 (33.3)		30 (66.7)	15 (33.3)	
34-41	17 (42.5)	23 (57.5)		24 (60.0)	16 (40.0)		24 (60.0)	16 (40.0)	
42-50	10 (52.6)	9 (47.4)		13 (68.4)	6 (31.6)		13 (68.4)	6 (31.6)	
> de 50	5 (62.5)	3 (37.5)		5 (62.5)	3 (37.5)		5 (62.5)	3 (37.5)	
Marital status									
Not married	28 (53.8)	24 (46.2)	0.095	36 (69.2)	16 (30.8)	0.494	36 (69.2)	16 (30.8)	0.805
Married	28 (42.4)	38 (57.6)		45 (68.2)	21 (31.8)		45 (68.2)	21 (31.8)	
Divorced	3 (21.4)	11 (78.6)		9 (64.3)	5 (35.7)		9 (64.3)	5 (35.7)	
Widower	0 (0.0)	1 (100.0)		0 (0.0)	1 (100.0)		0 (0.0)	1 (100.0)	
Years of current residence, years									
< 1	2 (22.2)	7 (77.8)	0.434	7 (77.8)	2 (22.2)	0.670	7 (77.8)	2 (22.2)	0.044
1–5	15 (48.4)	16 (51.6)		22 (71.0)	9 (29.0)		22 (71.0)	9 (29.0)	
6–10	13 (56.5)	10 (43.5)		17 (73.9)	6 (26.1)		17 (73.9)	6 (26.1)	
11–15	3 (37.5)	5 (62.5)		6 (75.0)	2 (25.0)		6 (75.0)	2 (25.0)	
> 15	26 (41.9)	36 (58.1)		38 (61.3)	24 (38.7)		38 (61.3)	24 (38.7)	
Children									
No	33 (48.5)	35 (51.5)	0.322	50 (73.5)	18 (26.5)	0.139	50 (73.5)	18 (26.5)	0.383
Yes	26 (40.0)	39 (60.0)		40 (61.5)	25 (38.5)		40 (61.5)	25 (38.5)	
Number of children									
None	34 (48.5)	34 (51.5)	0.478	50 (74.6)	17 (25.4)	0.211	50 (74.6)	17 (25.4)	0.602
1-2	23 (34.8)	34 (51.5)		35 (61.4)	22 (38.6)		35 (61.4)	22 (38.6)	
> 2	3 (4.5)	6 (9.09)		5 (55.6)	4 (44.4)		5 (55.6)	4 (44.4)	

ICU = intensive care units.

Regarding the professional characteristics of the intensivists participating in the study and their relationship with the three dimensions of burnout syndrome, depersonalization was manifested as low mean values in professionals without specialization (Table 2).

**Table 2:** Characteristics of ICU workers according to dimensions and levels of burnout syndrome (n = 133)

Variables	Dimensions and levels of burnout syndrome								
	Emotional exhaustion			Depersonalization			Personal accomplishment		
	Low n (%)	High n (%)	p-value	Low n (%)	High n (%)	p-value	Low	High n (%)	p-value
Years of formation									
< 1	1 (50.0)	1 (50.0)	0.725	1 (50.0)	1 (50.0)	0.193	1 (50.0)	1 (50.0)	0.029
1–5	22 (51.2)	21 (48.8)		34 (79.1)	9 (20.9)		34 (79.1)	9 (20.9)	
6–10	15 (45.5)	18 (54.5)		20 (60.6)	13 (39.4)		20 (60.6)	13 (39.4)	
11–15	11 (42.3)	15 (57.7)		19 (73.1)	7 (26.9)		19 (73.1)	7 (26.9)	
> 15	10 (34.5)	19 (65.5)		16 (55.2)	13 (44.8)		16 (55.2)	13 (44.8)	
Years of ICU									
< 1	6 (30.0)	14 (70.0)	0.380	13 (65.0)	7 (35.0)	0.435	13 (65.0)	7 (35.0)	0.325
1–5	28 (45.2)	34 (54.8)		46 (74.2)	16 (25.8)		46 (74.2)	16 (25.8)	
6–10	18 (51.4)	17 (48.6)		22 (62.9)	13 (37.1)		22 (62.9)	13 (37.1)	
11–15	6 (40.0)	9 (60.0)		8 (53.3)	7 (46.7)		8 (53.3)	7 (46.7)	
> 15	1 (100)	0 (0.0)		1 (100)	0 (0.0)		1 (100)	0 (0.0)	
Shift									
Morning	2 (33.3)	4 (66.7)	0.577	6 (100)	0 (0.0)	0.028	6 (100)	0 (0.0)	0.503
Evening	1 (20.0)	4 (80.0)		5 (100)	0 (0.0)		5 (100)	0 (0.0)	
Night	14 (50.0)	14 (50.0)		18 (64.3)	10 (35.7)		18 (64.3)	10 (35.7)	
Integral	42 (44.7)	52 (55.3)		61 (64.9)	33 (35.1)		61 (64.9)	33 (35.1)	
Weekly working hours									
20 to 40	13 (43.3)	17 (56.7)	0.420	21 (70.0)	9 (30.0)	0.274	21 (70.0)	9 (30.0)	0.623
> 40	46 (44.7)	57 (55.3)		69 (67.0)	34 (33.0)		69 (67.0)	34 (33.0)	
Hours of rest on duty									
0-1	14 (40.0)	21 (60.0)	0.761	26 (74.3)	9 (25.7)	0.219	25 (74.3)	9 (25.7)	0.674
1-2	24 (48.0)	26 (52.0)		36 (72.0)	14 (28.0)		36 (72.0)	14 (28.0)	
> 2	21 (43.8)	27 (56.3)		28 (58.3)	20 (41.7)		28 (58.3)	20 (41.7)	
Specialization									
No	42 (49.4)	43 (50.6)	0.119	65 (76.5)	20 (23.5)	0.004	65 (76.5)	20 (23.5)	0.722
Yes	17 (53.4)	31 (64.6)		25 (52.1)	23 (47.9)		25(52.1)	23 (47.9)	
Type of specialization									
None	42 (49.4)	43 (50.6)	0.119	65 (76.5)	20 (23.5)	0.017	65 (76.5)	20 (23.5)	0.333
Lato sensu	17 (37.0)	29 (63.0)		24 (52.2)	22 (47.8)		24 (52.2)	22 (47.8)	
Stricto sensu	0 (0.0)	2 (100.0)		1 (50.0)	1 (50.0)		1 (50.0)	1 (50.0)	
Worked in other ICUs									
No	39 (45.3)	47 (54.7)	0.756	58 (65.2)	31 (34.8)	0.381	63 (73.3)	23 (26.7)	0.901
Yes	20 (42.6)	27 (57.4)		32 (72.7)	12 (27.3)		27 (57.4)	20 (42.6)	

ICU = intensive care units.

Table 3 shows the characteristics of responses from health workers as well as the dimensions of burnout. Higher levels of occupational stress in the emotional exhaustion dimension were more common in professionals who consumed alcohol at least twice a week. In addition, ICU professionals who used stimulants had higher levels of burnout. Regarding the depersonalization dimension, low levels of burnout were found among those who did not consume alcoholic beverages. Respondents who drank alcohol once or twice a week had high levels of burnout. Those who did not consume stimulants had lower levels of burnout.

**Table 3:** Lifestyle of ICU workers according to dimensions and levels of burnout syndrome

Variables	Dimensions and levels of burnout syndrome								
	Emotional exhaustion			Depersonalization			Personal accomplishment		
	Low n (%)	High n (%)	p-value	Low n (%)	High n (%)	p-value	Low	High n (%)	p-value
Physical activity									
No	30 (42.9)	40 (57.1)	0.713	49 (70.0)	21 (30.0)	0.545	49 (70.0)	21 (30.0)	0.336
Yes	29 (46.0)	34 (54.0)		41 (65.1)	22 (34.9)		41 (65.1)	22 (34.9)	
Weekly physical activity									
None	30 (42.9)	40 (57.1)	0.790	49 (70.0)	21 (30.0)	0.552	49 (70.0)	21 (30.0)	0.345
1–2	5 (41.7)	7 (58.3)		8 (66.7)	4 (33.3)		8 (66.7)	4 (33.3)	
2–4	16 (43.2)	21 (56.8)		22 (59.5)	15 (40.5)		22 (59.5)	15 (40.5)	
> 4	8 (57.1)	6 (42.9)		11 (78.6)	3 (21.4)		11 (78.6)	3 (21.4)	
Diet balanced									
No	24 (41.4)	34 (58.6)	0.543	41 (70.4)	17 (29.3)	0.513	41 (70.7)	17 (29.3)	0.130
Yes	35 (46.7)	40 (53.3)		49 (65.3)	26 (34.7)		49 (65.3)	26 (34.7)	
Hours of sleep at home									
< 6	19 (51.4)	18 (48.6)	0.600	27 (73.0)	10 (27.0)	0.230	27 (73.0)	10 (27.0)	0.003
6–8	14 (41.2)	20 (58.8)		19 (55.9)	15 (44.1)		19 (55.9)	25 (44.1)	
> 8	26 (41.9)	36 (58.1)		44 (71.0)	18 (29.0)		44 (71.0)	18 (29.0)	
Recreation									
No	14 (41.2)	20 (58.8)	0.665	24 (70.6)	10 (29.4)	0.673	24 (70.6)	10 (29.4)	0.363
Yes	45 (45.5)	54 (54.5)		66 (66.7)	33 (33.3)		66 (66.7)	33 (33.3)	
Alcoholic									
No	43 (54.4)	36 (45.6)	0.005	61 (77.2)	18 (22.8)	0.004	61 (77.2)	18 (22.8)	0.015
Yes	16 (29.6)	38 (70.4)		29 (53.7)	25 (46.3)		29 (53.7)	25 (46.3)	
Frequency of beverages									
Do not consume	42 (53.8)	36 (46.2)	0.027	60 (76.9)	18 (23.1)	0.025	60 (76.9)	18 (23.1)	0.010
1–2 times	15 (30.0)	35 (70.0)		27 (54.0)	23 (46.0)		27 (54.0)	23 (46.0)	
3 or more times	2 (40.0)	3 (60.0)		3 (60.0)	2 (40.0)		3 (60.0)	2 (40.0)	
Smoker									
No	59 (45.0)	72 (55.0)	0.124	89 (67.9)	42 (32.1)	0.603	89 (67.9)	42 (32.1)	0.168
Yes	0 (0.0)	2 (100.0)		1 (50.0)	1 (50.0)		1 (50.0)	1 (50.0)	
Caffeine or stimulants									
No	26 (56.5)	20 (43.5)	0.040	39 (84.8)	7 (15.2)	0.002	39 (84.8)	7 (15.2)	0.388
Yes	33 (37.9)	54 (61.2)		51 (58.6)	36 (41.4)		51 (58.6)	36 (41.4)	
Frequency of stimulants (week)									
Do not consume	25 (55.6)	20 (44.4)	0.236	38 (84.4)	7 (15.6)	0.003	38 (84.4)	7 (15.6)	0.571
1–2	12 (42.9)	16 (57.1)		20 (71.4)	8 (28.6)		20 (71.4)	8 (28.6)	
2–4	9 (42.9)	12 (57.1)		14 (66.7)	7 (33.3)		14 (66.7)	7 (33.3)	
> 4	13 (33.3)	26 (66.7)		18 (46.2)	21 (53.8)		18 (46.2)	21 (53.8)	
Drugs									
No	58 (45.0)	71 (55.0)	0.415	89 (69.0)1	40 (31.0)	75	89 (69.0)	40 (31.0)	0.587
Yes	1 (2once or twice 5.0)	3 (5.0)		(25.0)	3 (75.0)		1 (25.0)	3 (75.0)	

ICU = intensive care units.

Table 4 shows the mean values of inflammatory markers (IL-6 and IL-10) and oxidative stress markers (carbonyl and TBARS) in relation to the dimensions and levels (low and high) of burnout syndrome in ICU workers. There were no statistically significant signs of inflammation in any category between the levels of burnout syndrome and its dimensions. However, there was a positive relationship between the development of burnout syndrome and reduced personal performance, which was associated with oxidative lipid damage (TBARS) (p < 0.05).

**Table 4:** Levels of inflammation markers (IL-6 and IL-10) and oxidative stress (TBARS and CARBONYL) according to the dimensions of the burnout syndrome in ICU workers (n = 133)

Variables	Emotional exhaustion mean ± SD			Depersonalization mean ± SD			Personal accomplishment mean ± SD		
	Low	High	p-value	Low	High	p-value	Low	High	p-value
IL6	380.377 ± 250.039	419.405 ± 410.021	0.697	413.152 ± 403.133	379.704 ± 192.220	0.575	386.063 ± 367.899	428.814 ± 315.115	0.200
IL10	757.009 ± 587.101	886.068 ± 884.210	0.357	831.090 ± 872.482	825.780 ± 494.578	0.365	799.799 ± 817.968	877.841 ± 683.301	0.360
TBARS	0.031 ± 0.038	0.024 ± 0.041	0.213	0.030 ± 0.047	0.020 ± .016	0.329	0.027 ± 0.048	0.026 ± 0.020	0.015
Carbonyl	0.003 ± 0.006	0.005 ± 0.008	0.230	0.004 ± 0.009	0.004 ± 0.004	0.541	0.004 ± 0.009	0.004 ± 0.005	0.978

ICU = intensive care units; IL = interleukin; SD = standard deviation; TBARS = thiobarbituric acid reactive substances.

Table 5 shows the mean values of inflammatory markers (IL-6 and IL-10) and oxidative stress markers (carbonyl and TBARS) in relation to the dimensions of burnout syndrome by professional category. Regarding the values of inflammatory and oxidative stress markers by category, there were statistically significant changes (p < 0.05) for IL-6, IL-10, and carbonyl. When comparing professional categories (pairwise evaluation using Kruskal-Wallis test), nurses had high mean values of IL-10 (1380.781 ± 700.096; p < 0.05) compared with physicians (823.850 ± 878.225; p < 0.05) and physical therapists (503.530; ± 144.804; p < 0.05). This suggests that nurses in this sample had higher mean levels of inflammatory markers, indicating a greater likelihood of becoming ill. Regarding the protein damage marker, the greatest changes in carbonyl were observed in nurses (0.009 ± 0.06, p < 0.05) compared with physicians (0.004 ± 0.008, p < 0.05), nursing technicians (0.03 ± 0.008, p < 0.05), and physical therapists (0.002 ± 0.003, p < 0.05).

**Table 5:** Inflammation markers (IL-6 and IL-10) and oxidative stress markers (TBARS and carbonyl) by professional category (n = 133)

Professional category	IL6	IL10	TBARS	Carbonil
	Mean ± SD	Mean ± SD	Mean ± SD	Mean ± SD
Nurses	534.482 ± 319.036	1380.781 ± 700.096*	0.016 ± 0.012	0.009 ± 0.006*
Physical therapists	288.353 ± 82.681	503.530 ± 144.804†	0.014 ± 0.004	0.002 ± 0.003†
Speech therapists	268.448 ± 145.260	579.039 ± 167.141	0.013 ± 0.002	0.006 ± 0.005
Physicians	318.082 ± 95.437	625.618 ± 455.797†	0.027 ± 0.047	0.004 ± 0.008†
Nursing technicians	422.732 ± 427.058	823.850 ± 878.225†	0.033 ± 0.045	0.003 ± 0.008†
p-value	0.035	0.000	0.058	0.002

* ^†^Different symbols in the columns indicate significant difference

IL = interleukin; SD = standard deviation; TBARS = thiobarbituric acid reactive substances.

## DISCUSSION

Analysis of burnout syndrome and the levels of its three dimensions revealed that the nursing staff presented the most relevant statistical information. Nurses showed high levels of emotional exhaustion. In a literature review of publications from 2010 to 2015 in three databases, Moraes Filho & Almeida^17^ found that nursing, especially in services of high and medium complexity and in ICUs, presented a higher level of occupational stress.

From the literature, burnout has several variables that can indicate the development of the syndrome. There is no reference to training time and experience, but some sectors and job categories are more conducive to psychological stress, especially professional training at the beginning of a career. This is because of the need to make critical decisions, the challenges of securing a position in the labor market, and the guarantees of well-being and financial security.^18,19,20^

Our study showed low levels of reduced personal accomplishment in professionals aged over 21 years and those who had been residing in the municipality between 1 and 5 years. The variables of age and duration of work are controversial; however, our results, in agreement with previous studies that argue that burnout syndrome can be found in professionals with more labor market experience, because there is a decline in their health, physical limitations, saturation and exhaustion due to long-term work in the same field.^21,22^

According to Kluger et al.,^23^ stress levels may be related to involvement in different hospitals and the workload required of health care professionals. A study with health care professionals from the neonatal ICUs found that working in intensive care generates strong affective involvement. If the health worker does not know how to manage this involvement with professional routines, feelings of disappointment with the service can arise, which can worsen the human relationship with patients and lead to increased stress due to workload.^24^

A study with 7,288 U.S. physicians divided into three *OK* groups based on the length of their careers (early career [0–10 years], mid-career [11–20 years], and late career [over 20 years]) found a prevalence of burnout among mid-career physicians, who corresponded to approximately 60% of the sample. They expressed greater frustration with their choice of specialty and the impact on their personal lives.^25^

The low work relationship dimension of the syndrome reveals factors such as professionals’ low interest in the development of their work. Lower scores in this dimension indicate a high likelihood of burnout syndrome.^13^ In addition to presenting the results related to job stress when considering those with longer service (related to their length of stay), other studies have also shown that professionals working in other ICUs have high levels of burnout syndrome, confirming the overload of work activities due to time constraints.

The respondents in this study showed high levels of burnout on the depersonalization dimension, especially among those who were in their first year of training and those who reported having at least one lato sensu specialization in intensive care, either through postgraduate or residency programs. A study by Nascimento Sobrinho et al.,^26^ of more than 300 Brazilian physicians working in ICUs found that the prevalence of burnout was lower among physicians who did not have a specialization in intensive care. In a systematic review and meta-analysis of burnout among French physicians, Ziad et al.^27^ reported that the highest rates of professional exhaustion were found among specialists, especially those with residency training.

Sleep and lifestyle are directly related to quality of life. Professionals who reported getting less than 8 hours of sleep per night showed statistically significant results indicating high levels of burnout in the low personal accomplishment dimension. These findings reflect the reality of healthcare professionals, especially nurses and physicians, who often do not get enough sleep. It is recommended that adults get 7-8 hours of sleep per night; sleep deprivation can lead to demotivation, cognitive deficits, reduced professional effectiveness, and impaired quality of life.^27,28^

Observation of the lifestyle of the study sample revealed a propensity for high levels of burnout syndrome in terms of emotional exhaustion among professionals who consumed alcohol and those who consumed stimulants (caffeine, tea, or soda). In the depersonalization dimension, we found that most professionals, especially those who did not consume alcohol, had a low propensity for burnout. Alcohol consumption causes disorders that result in significant impairments in social and occupational domains. For example, alcohol consumption by health care workers can affect their skills and the safety of the procedures they perform. This can lead to psychological problems that may develop into psychiatric disorders or professional burnout, thereby increasing the risk of errors in care.^29^

An international study investigated the relationship between dimensions of burnout syndrome, depersonalization and emotional exhaustion, alcohol and fast-food consumption, physical activity, and self-medication among 2,623 professionals working in university hospitals in Portugal, Greece, Romania, Bulgaria, Turkey, Macedonia, and Croatia. The study found that one in five health care professionals had high burnout scores, which were significantly associated with fast food consumption, lack of exercise, use of analgesics, and more frequent alcohol consumption.^10^

A study on the risks of alcohol consumption among Danish physicians in relation to burnout syndrome showed high levels of burnout among professionals who consumed alcohol. Depersonalization was the dimension most emphasized in this study.^30^

According to Shirom,^31^ when faced with a problematic situation, some people engage in health-damaging behaviors as a coping strategy to alleviate distress in the short term. This type of behavior can act as a potentiator of the mechanisms that develop burnout syndrome.^32^ The behavioral process related to occupational stress can be seen as part of a situation underlying the damage caused by burnout. This damage is caused by work stressors and acts as a mental and physical escape mechanism that can intensify the development of burnout and other health problems.^33,34^

This study also sought to correlate the values of oxidative stress and inflammation markers with the dimensions of burnout syndrome. Oxidative stress is evaluated through markers that identify and quantify the imbalance of the antioxidant action overcome by the production of ROS, which favor the oxidation of biomolecules and generate specific metabolic products mainly derived from the oxidation of lipids, proteins, and deoxyribonucleic acids.^35^ The greatest expression of oxidative damage occurs in lipids and proteins, and in this study, carbonyl and TBARS were used. IL-6 and IL-10 inflammation markers were also evaluated. During the collection of information and blood samples for the study, none of the participants reported being ill or showed visible signs of pathologies.

When the dimensions of burnout syndrome were cross-checked with the mean values of inflammatory markers (IL-6 and IL-10) and oxidative stress (carbonyl) among ICU professionals, no statistical evidence was obtained when using the collectively assessed mean values. However, a significant correlation between the development of burnout syndrome and oxidative stress

was observed for TBARS. Our study identified mean TBARS values in the burnout syndrome dimension of reduced personal accomplishment, indicating a relationship between occupational stress and oxidative stress for this marker. Therefore, the results suggest a relationship between oxidative stress and occupational stress in ICU workers with more experience and longer working hours in this sector, confirming data from similar studies in Spanish emergency health professionals.^30,31,32^

When categorized, inflammatory markers and oxidative protein markers (carbonyl) showed differences between the categories. Significant differences between nurses and physical therapists and between nurses and physicians were found in the pairwise evaluation using the Kruskal-Wallis test for IL-10 and carbonyl levels. The mean values of IL-10 and carbonyl indicated that nurses had the highest levels of inflammation and protein damage, respectively, compared to the other categories that stood out. There were no significant changes in the other categories.

The biomarker for the lipid peroxidation product is malondialdehyde (MDA), which is a derivative of the endocyclization breakdown of polyunsaturated fatty acids, such as linoleic, arachidonic, and docosahexaenoic acids.^36,37,38^ For the ICU professionals surveyed, the mean values of MDA were identified in the dimension of low personal involvement with work, which refers to burnout syndrome, characterizing the relationship between occupational stress and oxidative stress for this marker.

These data suggest that the inflammatory response was present in the study population, but there was no significant difference in IL-6 and IL-10 levels because the values were averaged over the entire sample. This can be considered a limitation of the study, since only the collection and measurement of the markers was performed, and we did not have data to compare the progression of possible illnesses. It can be affirmed that nurses presented higher mean values of IL-10 and carbonyl, which were statistically significantly (p < 0.05) higher than those of physicians, physical therapists, and nursing technicians. This indicates a greater inflammatory response and increased oxidative stress in nurses. Consequently, there appears to be a greater potential for the development of morbidities due to increased oxidative stress.

ICU workers have a high propensity to develop burnout syndrome. Among the three dimensions of the syndrome, low personal involvement in work was related to oxidative stress, as indicated by changes in mean TBARS levels in the study population. These aspects were particularly pronounced among professionals associated with ICUs, showing that a longer duration of work is associated with greater stress-inducing stimuli, leading to adverse effects on the physical and mental health of these intensivists.

## CONCLUSIONS

In conclusion, ICU workers have a strong propensity to develop burnout syndrome due to the stressors involved in their work activities. Among the three dimensions of the syndrome, low personal involvement in work was found to be associated with oxidative stress, as evidenced by changes in mean MDA levels. These findings indicate that the longer the duration of work, the greater the number of stress-inducing stimuli that have a significant impact on the physical and mental health of intensivists.

Finally, the results indicated that stress is associated with the environmental conditions of work, particularly the risks of oxidative stress. These environmental conditions pose significant risks to the health and safety of health care workers and affect their physical and mental well-being. Consequently, there is a need to establish measures to protect the health and lives of these professionals, thereby ensuring better overall health care.

### ETHICS APPROVAL

This study was approved by the Research Ethics Committee of UNESC and conducted in compliance with the criteria developed by the National Commission of Ethics in Research of Brazil, Resolution no. 510, of April 7, 2016 (certificate of ethical appraisal nº 61075716.4.0000.5062 and approval opinion nº 1.934.066).

### LIMITATIONS

The lack of significant differences between the inflammatory markers can be considered a limitation of the study, as only the collection and measurement of the markers were performed. We did not have data to compare the progression of possible illnesses.
